# The effect of faith in intuition on moral judgment: The mediating role of perceived harm

**DOI:** 10.3389/fpsyg.2022.1084907

**Published:** 2022-12-13

**Authors:** Shanshan Jiang, Shang Ding, Daoqun Ding

**Affiliations:** ^1^Business School, Central South University, Changsha, China; ^2^Department of Psychology, School of Education Science, Hunan Normal University, Changsha, China; ^3^Center for Mind and Brain Sciences, Hunan Normal University, Changsha, China

**Keywords:** faith, intuition, moral, judgment, perceived harm

## Abstract

This study aimed to explore the relationship between faith in intuition and moral judgment and the underlying mechanism among Chinese college students using a questionnaire and experimental method. The results showed that levels of faith in intuition predicted more moral wrongness regarding ambiguous hurtful behaviors than unambiguous ones. Additionally, the perceived harm mediated the effect of individuals’ levels of faith in intuition on moral wrongness regarding ambiguous harm behaviors but not regarding unambiguous harm behaviors. The results of this study provide empirical evidence on the relationship between faith in intuition and moral judgment in Chinese culture and have implications for future studies of moral judgments.

## Introduction

Intuitive processing involves rapid, often preconscious, intuitive feelings that are experienced as vague but compelling (Zhang et al., 2016). It is widely believed that intuitive processing affects moral judgments, values, and behavior. It is particularly relevant for condemning shocking but objectively harmless behaviors (ambiguous harmful behaviors; Schein and Gray, 2018). For example, if the judgments of such behaviors result from intuitive processing, individuals more inclined to rely on intuition are more likely to perceive these behaviors as wrong (Friesdorf et al., 2015; Alós-Ferrer and Hügelschäfer, 2016). However, few studies have examined how moral judgments are affected by individuals’ faith in intuition in Chinese culture. Therefore, this study intends to elucidate the relationship between faith in intuition and moral judgment, expanding the empirical research on this topic and exploring the possible mechanisms underlying individual differences in Chinese culture.

Previous studies have found that intuitive processing plays a significant role in moral judgment (Gray and Keeney, 2015; Heintzelman and King, 2016). Individuals’ reflective ability (low faith in intuition) can negatively predict the moral wrongness of ambiguous hurtful behaviors (Mastrogiorgio, 2015; Pennycook et al., 2016). Both moral foundations theory (Gray et al., 2012a,b; Zhang et al., 2016) and dyadic morality theory (Haidt and Graham, 2007; Zhan and Wu, 2019) acknowledge that the condemnation of ambiguous harmful behavior is intuitive (Zhang et al., 2016; Schein and Gray, 2018; Zhan and Wu, 2019). For example, if moral judgments of ambiguous hurtful behaviors result from intuitive processing, individuals with higher faith in intuition are more likely to perceive these behaviors as wrong. Considering individual intuitive differences in the context of moral judgment helps explain people’s faith in the intuition of moral judgment regarding ambiguous harmful behaviors (Schein and Gray, 2018). As it violates deeply held intuitions about maintaining social order, harmful behaviors involving ambiguity tend to evoke individual condemnation of the unconventional. Moreover, for unambiguous hurtful behaviors, we theorize that individuals who are more inclined to rely on intuition may be more inclined to rely on social norms to guide their morality. Whether hurtful behaviors are ambiguous or unambiguous, the behavior itself violates “binding” values that preserve social customs and order (Scherer et al., 2015; Schein et al., 2016). Thus, individuals who rely more on intuition will find both ambiguous and unambiguous hurtful behaviors more immoral.

Therefore, we believe that individuals’ levels of faith in intuition predict differences in moral judgments of harmful behaviors (both ambiguous and unambiguous). In other words, individuals with higher levels of faith in intuition are more likely to make critical moral judgments through intuitive responses.

Harm plays a significant role in moral cognition, and not harming others is considered the fundamental element of morality (Park et al., 2016). Consideration of harm moderates costly moral behavior (FeldmanHall et al., 2016). When participants were asked to list a morally wrong action, for conservatives and liberals, harm was the most prevalent factor when referring to morality (Hofmann et al., 2014). Anthropological research also demonstrates that the relationship between harm and morality is cross-culturally universal, and people will consistently believe that harm is wrong and a moral offense (Barrett et al., 2016).

Dyadic morality theory also regards harm as the core issue of moral judgments and holds that an individual’s view of harm dominates the moral judgments of behaviors (Haidt and Graham, 2007; Zhan and Wu, 2019). Individuals attempt to match a particular behavior to a module of harm when making moral judgments. This module is automatically activated to create appropriate moral condemnation based on the importance of harm to human social life (Schein and Gray, 2018). If an action is judged as morally wrong, it is assumed that harm is produced (Schein and Gray, 2015, 2018).

Dyadic morality theory emphasizes the perceived harm, which need not objectively exist; instead, it only needs to be perceived to affect moral judgment (Gray et al., 2012a,b). Even ambiguous harmful behaviors can activate individuals’ perceived harm and elicit moral judgments (Gray et al., 2014). For example, pornographic images (Wright et al., 2014) and flag-burning (Welch and Bryan, 2000) are ambiguous and harmful behaviors and are also considered immoral. Notably, dyadic morality theory suggests that perceived harm is a continuum of intuition; intuition influences moral judgments and involves harm. Therefore, we suggest that the perceived harm may explain the relationship between individuals’ faith in intuition and moral judgments, thus playing a mediating role.

**Figure 1:**
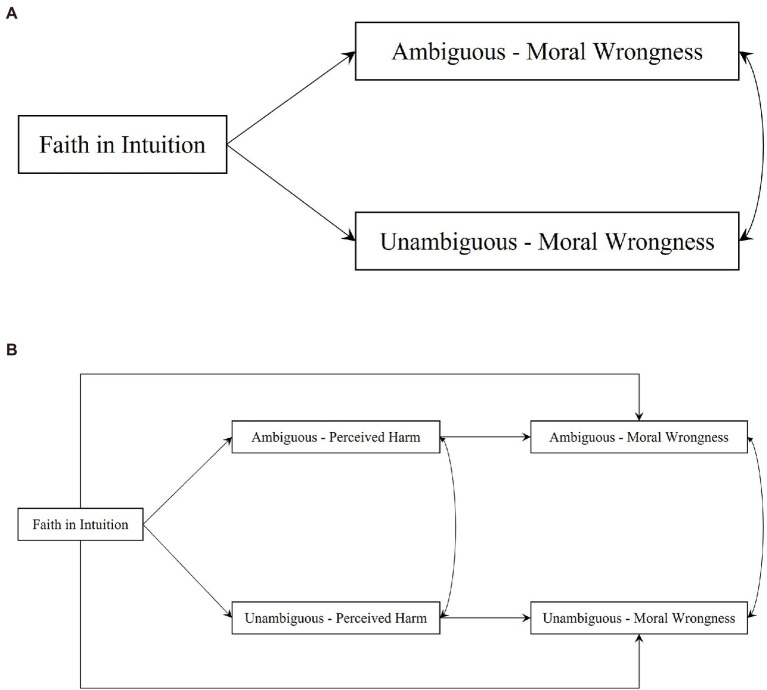
Theoretical model diagram. **(A)** Theoretical model diagram of Hypotheses 1a and 1b. **(B)** Theoretical model diagram of Hypotheses 2a and 2b.

To sum up, this study aimed to investigate the relationship between individuals’ faith in intuition and moral judgments and the mechanism of the perceived harm in this relationship in Chinese culture. Based on the literature review, we propose the following hypotheses (theoretical model diagram; see Figure 1).

*Hypothesis 1a*: Individuals’ levels of faith in intuition predict more moral wrongness regarding ambiguous hurtful behaviors.

*Hypothesis 1b*: Individuals’ levels of faith in intuition predict more moral wrongness regarding unambiguous hurtful behaviors.

*Hypothesis 2a*: Perceived harm will mediate the effect of individuals’ levels of faith in intuition on moral wrongness regarding ambiguous harmful behaviors.

*Hypothesis 2b*: Perceived harm will mediate the effect of individuals’ levels of faith in intuition on moral wrongness regarding unambiguous harmful behaviors.

This study tested these hypotheses through two experiments: Experiments 1 and 2 verified Hypotheses 1a and 1b and Hypotheses 2a and 2b, respectively.

## Experiment 1

### Participants

In total, 221 Chinese college students participated in Experiment 1. During the experiment, we set up one attention-check item (“This is an attention-check item; please select: somewhat agree”) adapted from Montal-Rosenberg and Moran (2022). Twelve participants failed this item and were excluded from the sample, leaving 209 participants (120 women, *M_age_* = 21.70, *SD_age_* = 1.596). The effective recovery rate was 94.57%. Before the experiment, all participants read and signed the informed consent and received compensation (10 RMB/approximately US $1.4) after completing the experiment. This experiment was approved by the ethics committee of our affiliated institution.

### Materials and methods

#### Faith in intuition

The Faith in the Intuition subscale of the Rational-Experimental Inventory was used for the measurement (Pacini and Epstein, 1999). The subscale includes seven items, for example, “I trust my initial feelings about people” and “Using my gut feelings usually works well for me in figuring out problems in my life.” The response options ranged from 1 (*completely disagree*) to 5 (*completely agree*). The higher the score, the higher the level of faith in intuition. The Cronbach’s alpha of faith in intuition in Experiment 1 was 0.765.

#### Moral judgment

Twenty-four items describing moral scenarios were used as moral judgment materials (Ward and King, 2018). Twelve items reflected ambiguous harmful behaviors (e.g., “Eat your pet dog after it was hit by a car and killed,” “Give your romantic partner a gift that was purchased for an ex,” “Have sex with a (dead) chicken and then eat it”), and another 12 items reflected unambiguous harmful behaviors (e.g., “Cheat on a romantic partner,” “Murder someone,” “Blame a coworker for your mistake”). Using E-prime, participants were randomly asked to rate the moral wrongness of these 24 items on a 7-point scale (“Do you think the action described is morally wrong?”). The higher the score, the stricter the moral judgment. The Cronbach’s alpha of moral wrongness in ambiguous and unambiguous harmful scenarios in Experiment 1 were 0.926 and 0.912, respectively.

### Results

#### Preliminary analysis

Table 1 presents the descriptive statistics and correlation analysis results of Experiment 1.^1^ Faith in intuition recorded a significant positive correlation with moral wrongness in ambiguous harmful scenarios (*r* = 0.172, *p* = 0.013). The correlation coefficient of moral wrongness in unambiguous harmful scenarios was not significant (*r* = 0.050, *p* = 0.476). The moral wrongness of ambiguous harmful scenarios recorded a significant positive correlation with that of unambiguous harmful scenarios (*r* = 0.379, *p* < 0.001).

**Table 1:** Descriptive statistics and correlation analysis results of Experiment 1.

	*M(SD)*	1	2	3	4	5
1. Faith in intuition	2.969 (0.583)	—				
2. Ambiguous—moral wrongness	5.615 (1.204)	0.172^*^	—			
3. Unambiguous—moral wrongness	6.111 (1.003)	0.050	0.379^***^	—		
4. Sex	—	−0.054	−0.010	−0.016	—	
5. Age	21.70 (1.596)	0.066	−0.050	0.071	−0.051	—

* *p* < 0.05;

*** *p* < 0.001.

M, mean; SD, standard deviation.

#### Model analysis

**Figure 2:**
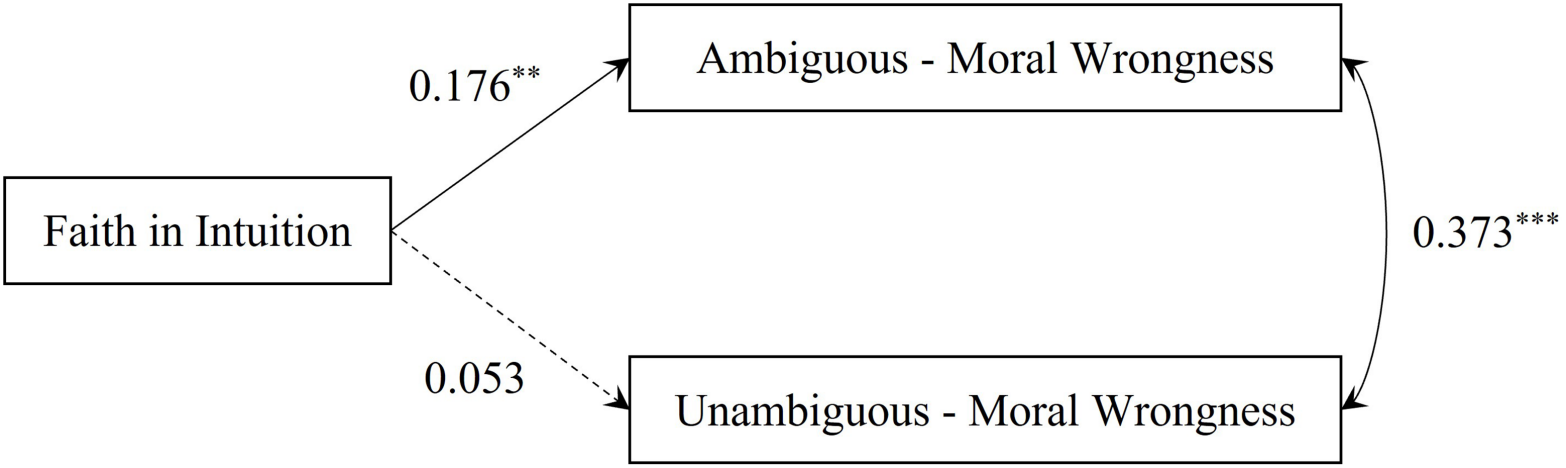
The model diagram of Experiment 1 (standardized results). ^**^p < 0.01, ^***^p < 0.001; dashed lines indicate non-significant coefficients.

All predictor variables were pooled for the collinearity test. The results showed that the variance inflation factors (VIFs) did not exceed two, indicating that Experiment 1 did not have a serious collinearity problem (Dormann et al., 2013). The model diagram of Experiment 1 is shown in Figure 2. It was found that faith in intuition could significantly positively predict moral wrongness in ambiguous harmful scenarios (β = 0.176, *p* = 0.009, 95% CI [0.044, 0.307]) after controlling for sex and age. However, the prediction of moral wrongness in unambiguous harmful scenarios was not significant (β = 0.053, *p* = 0.439, 95% CI [−0.082, 0.189]). Hypothesis 1a was verified, but Hypothesis 1b was not.

### Discussion

Experiment 1 showed that, in unambiguous harmful scenarios, faith in intuition had no significant impact on moral judgment; meanwhile, in ambiguous harmful scenarios, faith in intuition could significantly affect moral judgment. Individuals with higher faith in intuition made stricter moral judgments in ambiguous harmful scenarios in Chinese culture. The results verified Hypothesis 1a, indicating that the participants relied on intuition when making moral judgments in ambiguous harmful scenarios. Moreover, the levels of faith in intuition differed, affecting the strictness of moral judgments. This is consistent with previous findings (Ward and King, 2018).

## Experiment 2

In Experiment 1, the relationship between faith in intuition and moral judgment was preliminarily verified in Chinese culture. In Experiment 2, real-life moral scenarios faced by participants were more appropriate for further verifying the findings of Experiment 1. Additionally, Experiment 2 asked participants to rate the levels of harm in moral scenarios to explore how perceived harm plays a role in the relationship between faith in intuition and moral judgment.

### Participants

In total, 232 Chinese college students participated in Experiment 2. During the experiment, we set up one attention-check item, in which 24 participants failed and were excluded from the sample, leaving 208 participants (68 women, *M_age_* = 20.55, *SD_age_* = 4.749). The effective recovery rate was 89.66%. All participants read and signed the informed consent form and received compensation (15 RMB/approximately US $2.1) after completing the experiment. This experiment was approved by the ethics committee of our affiliated institution.

### Materials and methods

#### Faith in intuition

Faith in intuition was measured as in Experiment 1. The Cronbach’s alpha of faith in intuition in Experiment 2 was 0.716.

#### Moral judgment

The moral dilemmas presented by Gürçay and Baron (2017) were adapted to the Chinese cultural context. Experiment 2 used E-prime to ask participants to randomly rate 10 ambiguous harmful scenarios (e.g., “Lei Li was a doctor on duty in the emergency room when two dying patients were brought in. However, Li can only save one person at a time, and the other is likely to die. One is old and sick, and the other is young and healthy. If the young man survives, he may live for a long time; meanwhile, even if the older adult survives, he may not have much time left. Li could flip a coin and give them an equal chance of receiving treatment, but he chose to save the young man first because he had a longer life expectancy.” “Lei Li is using the internet to study depression among college students. To protect his participants’ privacy, he has agreed to encrypt them so that he does not know the identities of the people with depression. However, Li found that one of his participants had indicated on a questionnaire that he would commit suicide the next day. Although Li had promised to protect the participant’s privacy, he broke his promise by writing a computer program that identified the participant’s information and prevented his suicide”). The participants also randomly rated 10 unambiguous harmful scenarios (e.g., “Lei Li is an air traffic controller. A terrorist missile is heading for a jumbo jet with 500 passengers. If the missile hits the jet, all occupants will be killed. To prevent the airliner from being shot down, Li guided a small jet into the missile’s path and intercepted the missile; however, the small jet had 100 passengers on board, all of whom were killed.” “Lei Li is a health official. He learns that 10,000 people will die from a new strain of flu. Only one vaccine can suppress it; however, using it would kill 2,000 people. Li Lei used the new vaccine to save 8,000 people.”). A 7-point scale was used to score moral wrongness (“Do you think that the action described is morally wrong?”) and harm (“Do you think that the action described caused any harm?”). The higher the score, the stricter the moral judgment and the higher the level of perceived harm. Cronbach’s alpha of moral wrongness and the perceived harm in ambiguous and unambiguous harmful scenarios in Experiment 2 were all above 0.905.

### Results

#### Preliminary analysis

Table 2 presents the descriptive statistics and correlation analysis results of Experiment 2. Faith in intuition was significantly positively correlated with the moral wrongness of ambiguous harmful scenarios (*r* = 0.146, *p* = 0.035) but not with that of ambiguous harmful scenarios (*r* = 0.038, *p* = 0.589). Faith in intuition was significantly positively correlated with the perceived harm of ambiguous harmful scenarios (*r* = 0.173, *p* = 0.012); the perceived harm of unambiguous harmful scenarios correlation was not significant (*r* = 0.006, *p* = 0.931). The perceived harm of ambiguous harmful scenarios recorded a significant positive correlation with the moral wrongness of ambiguous harmful scenarios (*r* = 0.856, *p* < 0.001). The perceived harm of unambiguous harmful scenarios was significantly positively correlated with the moral wrongness of unambiguous harmful scenarios (*r* = 0.717, *p* < 0.001). The perceived harm of ambiguous harmful scenarios was significantly positively correlated with that of unambiguous harmful scenarios (*r* = 0.555, *p* < 0.001). The moral wrongness of ambiguous harmful scenarios was significantly positively correlated with that of unambiguous harmful scenarios (*r* = 0.643, *p* < 0.001).

**Table 2:** Descriptive statistics and correlation analysis results of Experiment 2.

	*M(SD)*	1	2	3	4	5	6	7
1. Faith in intuition	2.993 (0.512)	—						
2. Ambiguous—perceived harm	3.695 (1.233)	0.173^*^	—					
3. Unambiguous—perceived harm	3.308 (1.333)	0.006	0.555^***^	—				
4. Ambiguous-moral wrongness	3.622 (1.262)	0.146^*^	0.856^***^	0.475^***^	—			
5. Unambiguous—moral wrongness	3.562 (1.323)	0.038	0.510^***^	0.717^***^	0.643^***^	—		
6. Sex	—	0.121	0.003	−0.050	0.030	−0.010	—	
7. Age	20.55 (4.749)	0.019	0.032	0.034	0.057	0.033	0.103	—

M, mean; SD, standard deviation.

* *p* < 0.05;

*** *p* < 0.001.

#### Model analysis

The total effect of faith in intuition on moral wrongness was also tested in Experiment 2. After controlling for sex and age, the results showed that faith in intuition could significantly positively predict the moral wrongness of ambiguous harmful scenarios (β = 0.145, *p* = 0.034, 95% CI [0.011, 0.278]) after controlling for gender and age. However, the prediction of the moral wrongness of unambiguous harmful scenarios was not significant (β = 0.039, *p* = 0.574, 95% CI [−0.097, 0.176]). These results verified the stability of the findings of Experiment 1.

**Figure 3:**
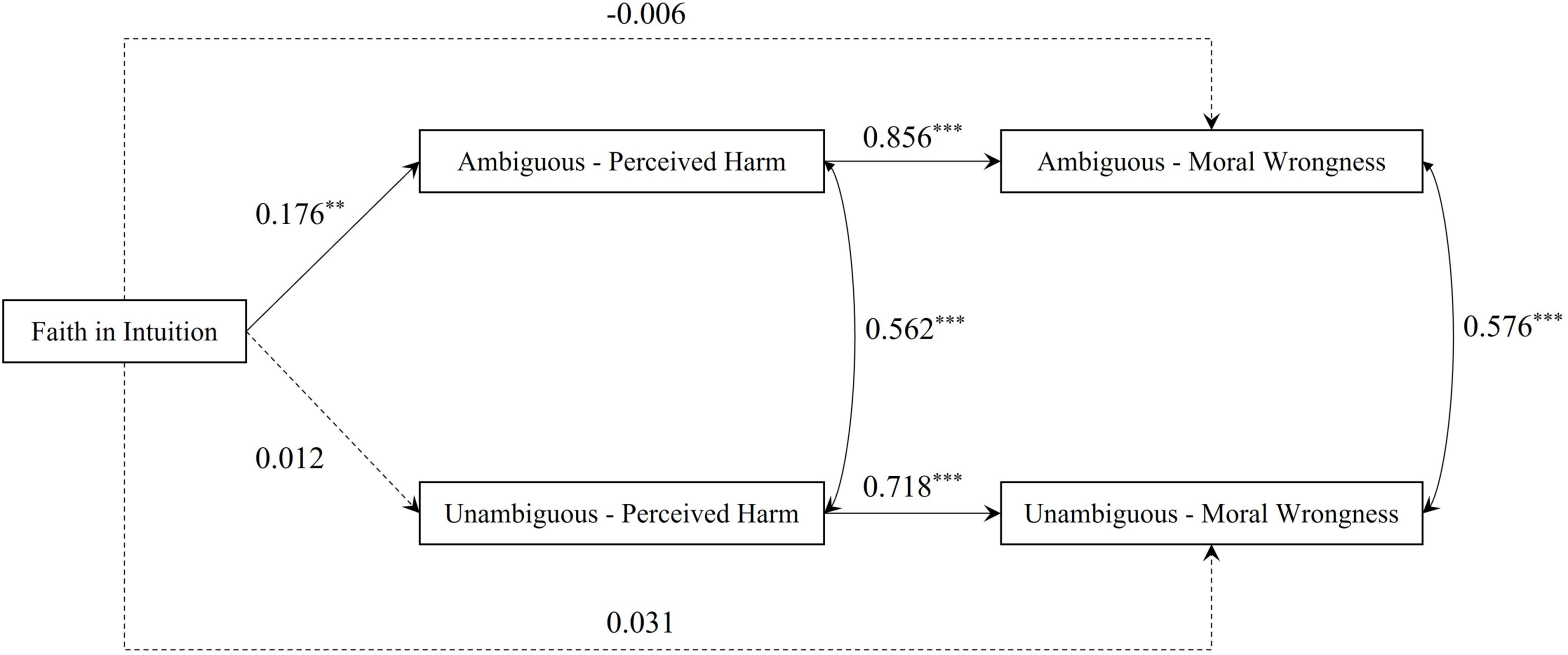
The model diagram of Experiment 2 (standardized results). ^**^p < 0.01, ^***^p < 0.001; dashed lines indicate non-significant coefficient.

All predictor variables were pooled for the collinearity test, and the results showed that the VIFs did not exceed two, indicating that Experiment 2 has no serious collinearity problem (Dormann et al., 2013). The model diagram of Experiment 2 is shown in Figure 3. The fitting results showed that the model was saturated, conforming to the standards. After controlling for sex and age, the results showed that faith in intuition could significantly and positively predict the perceived harm of ambiguous harmful scenarios (β = 0.176, *p* = 0.002, 95% CI [0.060, 0.285]) but not the perceived harm of unambiguous harmful scenarios (β = 0.012, *p* = 0.853, 95% CI [−0.120, 0.135]). The perceived harm of ambiguous harmful scenarios significantly positively predicted the moral wrongness in ambiguous harmful scenarios (β = 0.856, *p* < 0.001, 95% CI [0.718, 0.927]). The perceived harm of unambiguous harmful scenarios could also significantly and positively predict the moral wrongness of unambiguous harmful scenarios (β = 0.718, *p* < 0.001, 95% CI [0.552, 0.837]).

The bias-corrected bootstrap method (5,000 bootstrap samples) was used to test the mediating effect. The results showed that the perceived harm of ambiguous harmful scenarios played a significant mediating role between faith in intuition and moral wrongness (pathway: faith in intuition → perceived harm of ambiguous harmful scenarios → moral wrongness, estimate = 0.150, *SE* = 0.050, *p* = 0.003, 95%CI [0.051, 0.248]). However, the mediating role of unambiguous harmful scenarios was not significant (pathway: faith in intuition → perceived harm of unambiguous harmful scenarios → moral wrongness, estimate = 0.009, *SE* = 0.047, *p* = 0.854, 95% CI [−0.090, 0.096]). Hypothesis 2a was verified, whereas Hypothesis 2b was not.

### Discussion

The results of Experiment 2 showed that, when reading moral dilemmas for moral judgment, faith in intuition can still significantly affect moral judgment in ambiguous harmful scenarios, verifying the stability of the findings of Experiment 1. In addition, mediation analysis found that the perceived harm mediated the above relationship in ambiguous harmful scenarios. Compared to individuals with lower faith in intuition, individuals with higher faith in intuition perceived more harm and made stricter moral judgments in ambiguous harmful scenarios.

## General discussion

Intuitive information processing plays a significant role in contemporary moral psychology. However, few studies have explored the role of individual differences in relying on intuition in moral judgment in Chinese culture. This study investigated the relationship between faith in intuition and moral judgments in Chinese culture. The following conclusions emerged: First, individuals’ levels of faith in intuition predict greater moral wrongness regarding ambiguous hurtful behaviors but do not predict moral wrongness regarding unambiguous hurtful behaviors. Second, the perceived harm mediates the effect of individuals’ levels of faith in intuition on moral wrongness regarding ambiguous harm behaviors but not regarding unambiguous harm behaviors.

These results suggest that individuals more inclined to rely on intuition are more likely to condemn unethical behavior in ambiguous harmful scenarios than in unambiguous ones. This verifies Hypothesis 1a but not 1b. We theorize that this may be because the morality of the behavior is unclear in ambiguous harmful scenarios; therefore, individuals are more inclined to make moral judgments through intuition (Gray et al., 2014). In contrast, in unambiguous harmful scenarios, the action is immoral; therefore, individuals are more likely to use rational thinking rather than intuition to make judgments (Schein and Gray, 2018). This study suggests that individual differences in intuition are associated with attitudes toward certain types of behavior. Individuals who rely heavily on intuition condemn ambiguous harmful behaviors characterized by violations of social norms or conventions. Condemning ambiguous but harmful behavior is unsurprising; throughout human history, monitoring the social behavior of others and punishing those who do not conform to social norms and customs has been paramount. As a result, people are extremely sensitive to information that suggests they may be unstable or unreliable (Graham et al., 2009).

Experiment 2 showed that the perceived harm mediated the relationship between faith in intuition and moral judgment in ambiguous harmful scenarios and did not mediate the above relationship in unambiguous harmful scenarios. This verifies Hypothesis 2a but not 2b. This may be because, in ambiguous harmful scenarios, it is uncertain whether the behavior will cause harm and whether the individual will predict the actual harmful situation by intuition. However, in unambiguous harmful scenarios, the action is to ensure that a certain level of harm has been caused so that the individual need not use intuition to make predictions (Gray et al., 2012a,b; Schein and Gray, 2018).

Consistent with dyadic morality theory, these findings highlight the importance of harm in moral judgment (Ward and King, 2015). The relationship between perceived harm and moral judgment suggests that perceived harm is central to moral theory. The current research indicates that the moral judgment process of action requires attention to individual differences in intuition, moral value, and harm perception (Dienes, 2014; Gray et al., 2014). The moral foundations theory provides a rich value explanation for moral judgments and correctly anticipates the character and diversity of the moral landscape. However, previous studies have neglected to explain why people hold specific moral values (Pennycook et al., 2014; Royzman et al., 2015). Given a strong link between perceived harm and moral condemnation, it seems possible to understand why some people view behavior as harmful. This would elucidate significant individual differences in moral perception. Dyadic morality theory provides a comprehensive framework for understanding the cognitive processes that drive moral judgments.

Actions that evoke moral condemnation without actual harm are associated with primary motives, such as evaluating social relationships and avoiding pathogens. Intuitive processing has a long evolutionary history and is used by humans and animals. Owing to the holistic and associative nature of such thinking, intuitive processing may blur the line between actions that merely violate social conventions and those that involve harm, as both actions may reflect evolutionarily significant and long-term social and moral problems (Zhang et al., 2016). The moral condemnation of violations that lack apparent harm or victims is often interpreted as irrational and difficult to change. This study’s results provide further evidence that these ambiguous behaviors are condemned because they are considered harmful (Gray et al., 2012a; Baumard et al., 2013). It is difficult for individuals who rely strongly on intuition to change their intuitive perception of these situations as morally wrong.

The findings have theoretical implications for understanding the nature of faith in intuition and its role in moral judgment. Faith in intuition reflects the degree to which people rely on or prefer intuitive/empirical processing. Individuals with higher levels of faith in intuition experience more negative reactions to ambiguous harmful scenarios. The habitual tendency of individuals to trust their intuition may prompt them to focus more on such intuition; thus, faith in intuition involves not only a high reliance on intuitive processing but also a tendency to have a higher intuitive response to certain stimuli. A direction for future research is to explore whether the higher intuitive response experience of individuals with higher levels of faith in intuition applies to domains other than the moral domain. Additionally, the results of this study have certain practical significance, which may lead to a better understanding of moral judgments and reasons in life. It is helpful to deepen people’s understanding of their moral judgment and provide a new perspective for explaining moral judgment.

This study had several limitations. First, we measured individuals’ moral judgments by reading different moral scenarios influenced by the practice and social expectation effects (Ajzen, 1985, 1991). Future research could verify the findings of this study through field experiments. Second, this study showed a relationship between faith in intuition and moral judgments; however, it remains unclear whether individuals engage in similar behaviors. Future research could explore the relationship between faith in intuition and moral decision-making. Third, this study proposed perceived harm as a mediating variable based on dyadic morality theory. However, this is not unique. Future studies can also explore the relationship between faith in intuition and moral judgment from other perspectives, such as meaning in life (Heintzelman and King, 2016). Fourth, this study was conducted in the context of Chinese collectivist culture, which may be influenced by certain cultural factors. Future studies can further discuss the contents of this study from a cross-cultural perspective. Finally, studies can further explore the cognitive neural mechanisms of the relationship between faith in intuition and moral judgments through brain imaging.

## Data availability statement

The raw data supporting the conclusions of this article will be made available by the authors, without undue reservation.

## Ethics statement

The studies involving human participants were reviewed and approved by Ethics Committee of Hunan Normal University. The patients/participants provided their written informed consent to participate in this study.

## Author contributions

SJ was involved in the conceptualization, data analysis, and interpretation and wrote the manuscript. SD was involved in the data collection and data analysis. DD was involved in the manuscript editing, ethical approval, and interpretation. All authors contributed to the article and approved the submitted version.

## Funding

This paper was supported by the National Social Science Foundation of China (19BSH127).

## Conflict of interest

The authors declare that the research was conducted without any commercial or financial relationships that may be construed as a potential conflict of interest.

## Publisher’s note

All claims expressed in this article are solely those of the authors and do not necessarily represent those of their affiliated organizations, or those of the publisher, the editors and the reviewers. Any product that may be evaluated in this article, or claim that may be made by its manufacturer, is not guaranteed or endorsed by the publisher.

## Footnotes

^1^SPSS 25 was used for descriptive statistics and correlation analysis, and Mplus 8.3 was used for model and mediation analysis.
